# Mapping and modelling land degradation vulnerability in a semi-arid region: a case study from Battalgazi District, Turkiye

**DOI:** 10.7717/peerj.20606

**Published:** 2026-01-27

**Authors:** Miraç Kılıç

**Affiliations:** Department of Soil Science and Plant Nutrition, Malatya Turgut Özal Üniversitesi, Malatya, Battalgazi, Turkey

**Keywords:** Land degradation, Machine learning, Remote sensing, Semi-arid regions, Topographic indices, Vulnerability mapping, Explainable AI, Soil erosion

## Abstract

**Background:**

Land degradation threatens and the provision of ecosystem services worldwide. Land degradation vulnerability (LDV) assessments still lack the necessary spatial detail and predictive accuracy, and the integration of multiple spectral indices with machine learning remains underexplored. This study addresses the critical importance of spatially mapping vulnerability to land degradation and develops a novel framework that combines advanced machine learning and uncertainty measurement with the STORIE Index Rating (SIR), a semi-quantitative method for assessing potential soil productivity. This framework aims to spatially predict the vulnerability of soils in the study area to land degradation with high accuracy.

**Methods:**

This study addresses this gap by introducing HyStoRSM, a novel framework that integrates land-survey-derived data, remote sensing, and machine learning. This study presents a case study of the HyStoRSM framework in the Battalgazi district (940.5 km^2^) of Malatya province, which is representative of continental semi-arid conditions in the upper reaches of the Euphrates Basin in Eastern Anatolia. The framework integrates land survey data (major soil groups, land use capability, slope-depth combination, and erosion severity), spectral indices derived from Landsat 8 OLI/TIRS imagery, and topographic indices calculated from SRTM (Shuttle Radar Topography Mission) data. Landsat 8 and SRTM data from 2023 were processed on the Google Earth Engine platform. Local LDV scores were generated using the geometric mean form of the SIR. An extreme gradient boosting (XGBoost) regression model, optimized using Optuna, estimated continuous LDV scores, while SHapley Additive exPlanations (SHAP) provided insights into feature importance.

**Results:**

The optimized XGBoost regression model, with hyperparameters tuned using 5-fold cross-validation with Optuna-based hyperparameter optimization and validated on an independent 30% test dataset, achieved high prediction accuracy (*R*^2^ = 0.74, RMSE = 0.1285, MAE = 0.1002, and Huber Loss = 0.0083). SHAP analysis revealed that the length-slope factor was the most influential variable, followed by the stream power index and the Normalized Difference Vegetation Index (NDVI). These results demonstrated that hydro-topographic variables had a greater impact on LDV than spectral indices. Accordingly, an LDV map at 30 m spatial resolution was produced. Spatial analysis indicated that 21.7% and 20.3% of the study area exhibited high and very high LDV, primarily concentrated in the southern and southeastern regions. Conversely, low and very low vulnerabilities covered 16.9% and 12.4% of the area.

**Conclusions:**

The HyStoRSM framework integrates multisource satellite data, land survey data, and advanced machine learning into a single, interpretable framework. This enables proactive, precise land degradation risk management, especially in semiarid regions where terrain and hydrologic controls drive erosion vulnerability.

## Introduction

Land degradation threatens food security and ecosystem services, and is responsible for 85% of the global decline in soil quality and a 17% yield loss in crop production (Gebremichael, Gebremariam & Desta, 2025). This problem is further exacerbated in semi-arid regions, where the combination of shallow profile depth, high rainfall erosivity, and sparse vegetation cover often results in annual soil losses. Therefore, the development of reliable land degradation vulnerability (LDV) mapping systems has become an urgent priority for early detection and sustainable management of land degradation (Kaliraj et al., 2024).

In recent years, spectral indices derived from optical satellite imagery have enabled researchers to monitor land degradation across large areas at low cost. Missions such as Landsat-8 have made this monitoring capacity even more accessible, while radiometric indicators such as the Normalized Difference Vegetation Index (NDVI) have been widely used (Badapalli et al., 2025). This methodological advancement is particularly valuable in arid and semi-arid ecosystems, where conventional field-based soil monitoring approaches are constrained by spatial heterogeneity, accessibility limitations, and resource requirements. Recent developments in pedometric modeling demonstrate that ensemble machine learning techniques consistently achieve robust predictive performance for soil degradation processes, with studies reporting area under the curve (AUC) values ranging from 0.86 to 0.94 across diverse dryland pedo-environments (Badapalli et al., 2025; Khan et al., 2025; Roy & Saha, 2022; Temerdek-Ivan et al., 2025). These methodological innovations indicate that the synergistic application of advanced computational algorithms with satellite-derived spectral indices provides a scalable framework for quantifying soil erosion vulnerability and land degradation susceptibility in water-limited environments, thereby addressing critical knowledge gaps in arid land soil conservation and sustainable land management.

There are critical limitations in current approaches that constrain their effectiveness in comprehensive land degradation assessment. The dominant influence of topographic factors on erosion vulnerability cannot be adequately represented in most spectral-based models, as conventional remote sensing indices fail to capture subsurface soil constraints and microtopographic hydrology controllers that fundamentally drive degradation processes in arid environments (Conoscenti & Rotigliano, 2020). Satellite-derived spectral models demonstrate considerable limitations in characterizing subsurface soil attributes, with recent investigations acknowledging that subsurface property mapping still requires substantial methodological refinement despite achieving surface-level *R*^2^ values up to 0.72 (Mendes et al., 2019). The quantification of microtopographic features, where elevation variations often remain below one meter yet critically influence hydrological connectivity, presents a persistent data-intensive challenge that conventional satellite-based approaches cannot adequately resolve (Shukla et al., 2023). While topographic parameters are recognized as essential components of erosion modeling frameworks, comprehensive assessments require the integration of climatic variables, pedological properties, and land management practices, and these datasets are frequently unavailable due to resource constraints, particularly in developing arid regions (Huang et al., 2022). Furthermore, the reliability of spectral indices for quantitative soil assessment is compromised by methodological inconsistencies and a lack of standardization protocols, with diverse analytical approaches significantly constraining study comparability and result reproducibility (Chen et al., 2025b). Moreover, existing studies often focus on either field-based soil property assessments or multispectral remote sensing integrations, rarely integrating these two datasets at the same pixel scale (Luo et al., 2025). As a result, high spatial coverage machine learning maps ignore edaphic factors, while maps focusing on land survey data lose temporal relevance.

The three main limitations that exacerbate this gap are outlined below: First, site-derived edaphic parameters such as profile depth, composition, drainage class and erosion severity are not included in model inputs due to a lack of sufficient sample points (Oraegbu & Jolaiya, 2024). Second, validation of models is mostly limited to a single metric (ROC-AUC or *R*^2^), whşile cross-checking with independent field data and reporting multiple performance measures is not widespread (Badapalli et al., 2025). Third, reproducibility remains an issue as traditional grid search is used instead of systematic approaches in hyper-parameter optimization (Moradi et al., 2024). Therefore, an urgent need exists for a hybrid and interpretable modeling approach. Systematically combining land survey-derived edaphic layers (national field-survey map products) with multi-source satellite signals and hydro-topographic indicators can both improve prediction accuracy and produce transparent factor attribution maps for management policies.

In this study the HyStoRSM (land survey derived edaphic layers+ Remote Sensing + Machine Learning) framework was proposed to map the vulnerability of semi-arid lands to degradation. This study hypothesis that combining land survey derived edaphic layers (soil depth, large soil group, erosion severity) with topographic-hydrological indices and radiometric remote sensing data, and interpreting Optuna-optimized XGBoost models with SHapley Additive exPlanations (SHAP), will: (i) improve prediction accuracy beyond single-source reference values, and (ii) produce transparent factor attribution maps suitable for management scenarios.

The main objectives of this research are to: (i) integrate land survey data, spectral indices derived from Landsat 8 OLI/TIRS bands and Shuttle Radar Topography Mission (SRTM)-derived topographic layers; (ii) predict the continuous LDV score by optimizing the extreme gradient boosting (XGBoost) regression model with Optuna; and (iii) improve model explainability with SHAP; and (iv) produce 30 m resolution risk maps. The main innovations are: (1) integrating edaphic layers derived from national land survey products are integrated with multi-source satellite-derived indicators and hydro-topographic covariates in a novel, interpretable framework for LDV assessment (LDV); (2) ensuring reproducibility with Optuna-based systematic hyper-parameter optimization; (3) addressing the lack of transparency by quantifying the pixel-level effects of factors with SHAP outputs; and (4) ensuring the applicability of the method in other semi-arid regions with cloud-based code infrastructure. HyStoRSM aims to establish a new reference point for proactive land degradation risk management in water-limited environments.

## Materials & Methods

### Study area

**Figure 1:**
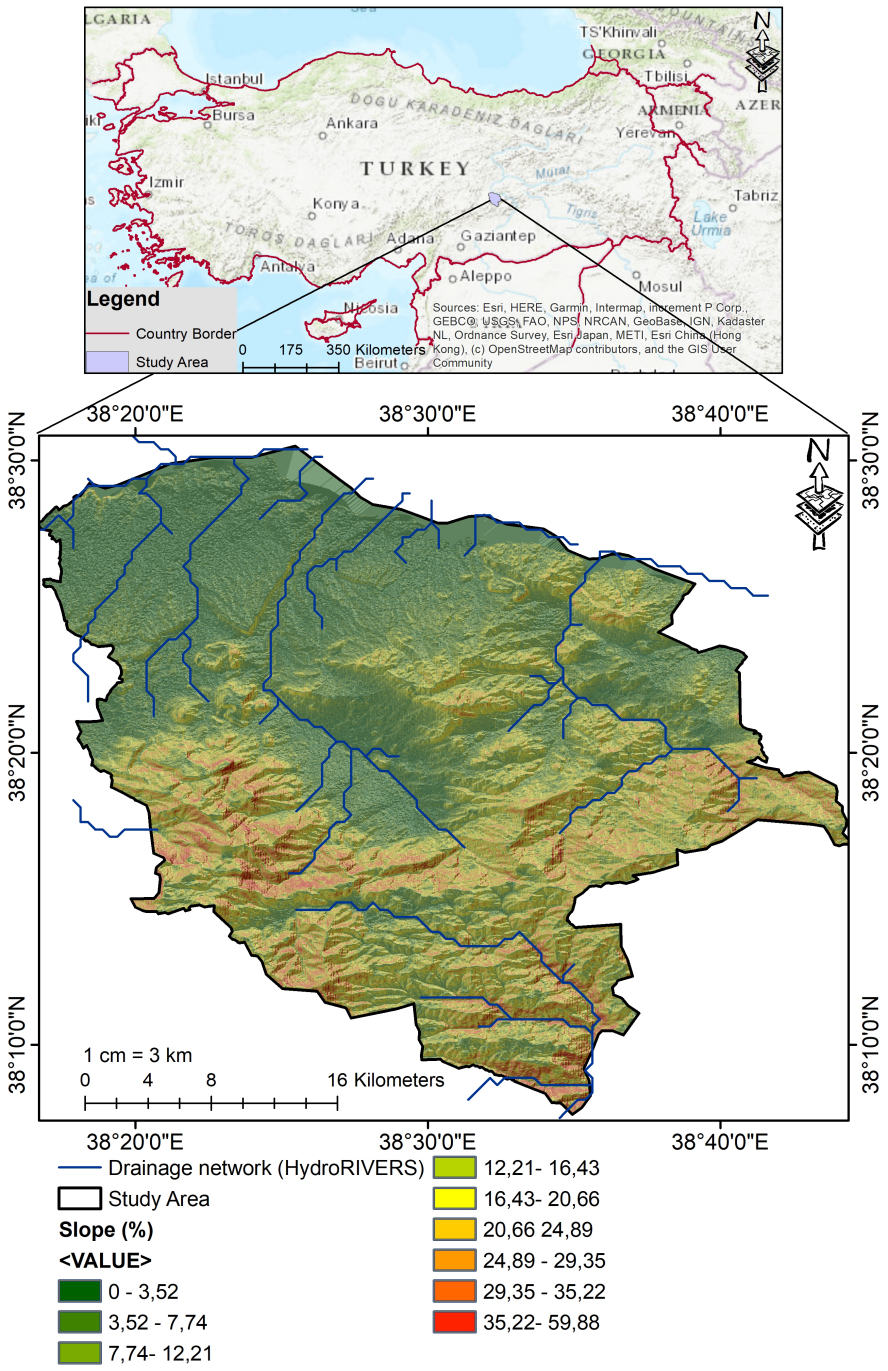
Geographic location of the study area. Topographic context and drainage network of the Battalgazi study area (Malatya, Türkiye). The main panel shows hillshade derived from the SRTM 30-m DEM with slope (%) classes computed from the same DEM (Farr et al., 2007). The drainage network is from HydroRIVERS (Lehner & Grill, 2013; Lehner, 2019). Administrative boundaries are from GADM v4 (GADM, 2022). The inset locates the study area using an ArcGIS basemap (Esri, Redlands, CA, USA).

The study area covers the administrative boundaries of Battalgazi district, located in the northwestern part of Malatya province, Turkiye (Fig. 1). The study area covers 940.5 km^2^ of land and is an important agro-ecological transition zone within the Upper Euphrates Basin of Eastern Anatolia. Surrounded by Yazıhan district to the north and the outskirts of Malatya city center to the south, the area is located between approximately 38°30′ and 38°45′ north latitude and 38°10′ and 38°30′ east longitude. The study area is covered by Neogene-aged sedimentary and volcanic units, mainly the Beylerderesi Formation. These formations consist of poorly bedded conglomerate, red-colored mudstone, and cross-bedded sandstone levels, and were fed by volcanic materials originating from basaltic trachyandesite (Tatar, 2016). The region experiences a semi-arid continental climate, characterized by long, dry summers and cold winters. Annual precipitation is around 380–450 mm, with significant year-to-year variability. Summer temperatures can exceed 40 °C and drop to −10 °C in winter. The annual sunshine duration, exceeding 2.700 h, increases water losses through evaporation and exacerbates soil moisture deficit (Arslan, 2006). Human impact has significantly transformed the natural vegetation (Kılıç & Günal, 2025). Although dry shrubs and perennial grasses were once the dominant feature of the steppe vegetation, most of these have been converted into agricultural fields and orchards. Apricot orchards, in particular, have expanded remarkably in recent years (Senturk, 2020). The soils are generally shallow to moderately deep and developed on clay-rich sediments of the Neogene. The dominant soil texture is loamy and clay loamy; smectite and illite are prominent clay minerals. The soils are neutral to slightly alkaline; salinity is generally low and organic matter content varies from low to medium, which makes the soils vulnerable to erosion, crusting, and degradation (Tatar, 2016).

### Land survey

**Figure 2:**
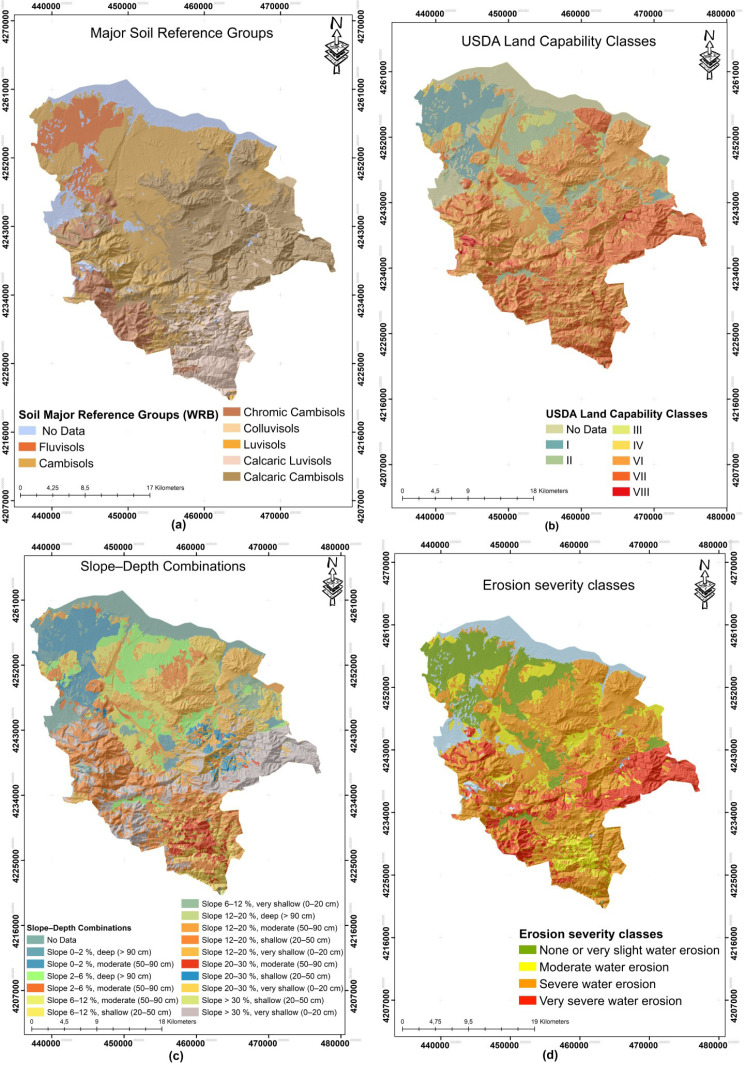
Land survey derived data. (A) Polygon colours denote WRB soil Major Reference Groups derived from the ESDAC European Soil Database v2.0 (https://esdac.jrc.ec.europa.eu/resource-type/datasets-list, CC-BY 4.0). (B) Map colours indicate USDA land capability classes (I–VIII). National Soil Information System (Ulusal Toprak Bilgi Sistemi, UTBS), Ministry of Agriculture and Forestry (T.C. Tarım ve Orman Bakanlığı), unclassified/public data (“tasnif dışı”), open data under national regulation (no specific international licence assigned) (C) Map colours show slope (% rise) and effective soil depth classes combined from the 30 m SRTM-v3 DEM (NASA/USGS; https://doi.org/10.5066/F7PR7TFT, public domain). Slope classes (0–2%, 2–6%, 6–12%, 12–20%, 20–30%, > 30%) were intersected with field-verified depth classes (very shallow 0–20 cm, shallow 20–50 cm, moderate 50–90 cm, deep > 90 cm). (D) Colours indicate water-erosion severity classes derived from the ESDAC RUSLE2015 Global Soil Erosion Map (https://esdac.jrc.ec.europa.eu/themes/rusle2015, CC-BY 4.0). None or very slight water erosion:(< 5 t ha^−1^ yr^−1^), Moderate water erosion: (5–10 t ha^−1^ yr^−1^), Severe water erosion: (10 –30 t ha^−1^ yr^−1^), Very severe water erosion: (> 30 t ha^−1^ yr^−1^).

The terrestrial data used include the Major Soil Groups (Fig. 2A), Land Use Capability (Fig. 2B), Slope-Depth Combination Classes (Fig. 2C) and Erosion Severity layers (Fig. 2D), which were prepared based on detailed soil surveys conducted throughout Turkiye. The Major Soil Groups classification is a pedologically based system used in Turkey’s soil inventory and is based on the 1938 soil classification system of the US Department of Agriculture. This traditional classification is different from the current Soil Taxonomy (Soil Survey Staff, 2014) and the FAO/UNESCO World Soil Map legend (FAO, 2006). The soil maps were produced by compiling 1:25.000 scale land surveys and subsequently published at 1:100.000 scale on a provincial basis. Land Use Capability Classification (LCC) is determined according to the standard classification that evaluates the suitability and limitations of land for agricultural production and basically follows the principles of USDA Land Capability Classification (LCC). This system grades lands from Class I to VIII, defining Class I land as areas with high production potential (with minimal constraints) and Class VIII as areas completely unsuitable for agricultural use (Helms, 1992). LCC data are based on detailed soil surveys conducted at 1:25.000 scale throughout Turkey and published as vector maps at 1:100.000 scale at the provincial level by the National Soil and Water Resources Information Center (Hallett et al., 2003). The classification follows USDA LCC principles and considers field-observed constraints including slope gradient, soil depth, stoniness, soil drainage, erosion hazard, and flooding risk (Klingebiel & Montgomery, 1961).

Erosion Severity data are layers that reflect the extent of erosion to which soils are exposed and include qualitative classes (*e.g.*, none/nearly none, moderate, severe, very severe erosion) based on field observations (Aksoy et al., 2010). The Erosion Severity layer was obtained from the national soil inventory compiled by the Ministry of Agriculture and Forestry, which reclassified the ESDAC RUSLE-based global annual soil loss raster (t ha^−^^1^ yr^−^^1^) into four classes (<5, 5–10, 10–30, and >30) and assigned the dominant class to Land Use Capability inventory polygons (Panagos et al., 2015; Borrelli et al., 2017). Slope-Depth Combination is an identification method that shows the topographic slope group and soil depth class of each map unit together; in this way, the physical limitations of the land (*e.g.*, steepness of the slope and effective depth of the soil) are evaluated together and their effects on land use are revealed (Anonymous, 2005). Slope gradient classes follow the national A-G classification system and effective soil depth classes are 0–20 cm, 20–50 cm, 50–90 cm, and ≥90 cm (measured to the root-limiting layer) (Ministry of Agriculture and Rural Affairs, 2008). Soil inventory polygons were reprojected to WGS 84/UTM Zone 37N; slope gradients derived from the digital elevation model were used for consistency validation, and the inventory-based Slope-Depth Combination was verified. All these vector datasets were produced by the National Soil and Water Resources Information Center (formerly the General Directorate of Rural Services, Ministry of Agriculture and Forestry) (Hallett et al., 2003). These datasets, classified using standard methodologies, were utilized as STORIE Index Rating (SIR) inputs in this study.

### Remote sensing data and image preprocessing

Landsat 8 OLI/TIRS (Operational Land Imager/Thermal Infrared Sensor) images and auxiliary data were utilized using the Google Earth Engine (GEE) cloud-based platform, which offers large data processing capacity and global scale data access (Gorelick et al., 2017; Geological Survey, 2021). First, the boundaries of Battalgazi district were determined as the study area and the geometry of the area was defined on GEE. A median composite image was created using Landsat 8 OLI and Thermal Infrared Sensor (TIRS) data from 2023. The images were taken from the Landsat 8 Collection 2 Level-2 dataset provided by the USGS (LANDSAT/LC08/ C02/T1_L2) and special masking processes were applied to minimize cloud, shadow, and saturation effects. Surface reflectance and surface temperature bands were scaled and made ready for analysis. Spectral indices for terrain degradation were calculated from the composite image (Table 1) following standard definitions and sources (Rouse et al., 1974; McFeeters, 1996; Xu, 2006; Wang & Qu, 2007; Khan et al., 2005; Gao, 1996; Guha & Govil, 2022; Zhang, Tang & Li, 2024).

**Table 1:** Formulas and descriptions of spectral indices calculated from Landsat 8 OLI/TIRS satellite. Formulas and sources of spectral indices derived from Landsat 8 OLI/TIRS imagery and used as covariates in the land degradation modeling framework. LST was computed using brightness temperature and surface emissivity corrections based on Band 10. All indices were resampled to 30 m resolution for spatial consistency. In the LST equation, TB is brightness temperature, K1 = 774.89, K2 = 1,321.08, *L*_*λ*_ = Top-of-Atmosphere (TOA) Radiance. Surface temperature with emissivity correction *λ* = 10.895 µm (Landsat 8 Band 10 center wavelength), *ρ* = *h*.*c*/*σ* = 1.438 × 10^−2^ m.K, *σ* = surface emissivity (Guha & Govil, 2022).

Index	Abbreviation	Formula	Source
Normalized Difference Vegetation Index	NDVI	$ \frac{{b}_{5}-{b}_{4}}{{b}_{5}+{b}_{4}} $	Rouse et al. (1974)
Normalized Difference Water Index	NDWI	$ \frac{{b}_{3}-{b}_{5}}{{b}_{3}+{b}_{5}} $	McFeeters (1996)
Modified Normalized Difference Water Index	MNDWI	$ \frac{{b}_{3}-{b}_{6}}{{b}_{3}+{b}_{6}} $	Xu (2006)
Normalized Multi-band Drought Index	NMDI	$ \frac{{b}_{5}- \left( {b}_{6}-{b}_{7} \right) }{{b}_{5}+ \left( {b}_{6}-{b}_{7} \right) } $	Wang & Qu (2007)
Normalized Difference Salinity Index	NDSI	$ \frac{ \left( {b}_{6}-{b}_{5} \right) }{{b}_{6}+{b}_{5}} $	Khan et al. (2005)
Land Surface Temperature	LST	${T}_{B}={K}_{2}\ln \left( {K}_{1}{L}_{\lambda }+1 \right) LST={T}_{B} \left( 1+ \left( \lambda \cdot {T}_{B}\rho \right) \cdot \ln \left( \right) \right) $	Guha & Govil (2022) and Zhang, Tang & Li (2024)
Normalized Difference Moisture Index	NDMI	$ \frac{{b}_{5}-{b}_{6}}{{b}_{5}+{b}_{6}} $	Gao (1996)

Within the scope of the multilayer analysis processes, a two-stage method was followed on the GEE platform. In the first stage, each band of Landsat 8 satellite imagery was processed separately, and multispectral spectral indices were calculated (Table 1).

All Landsat 8 OLI/TIRS Collection 2 Level 2 scenes intersecting the study area between 1 January and 31 December 2023, were compiled. A scene-level filter of CLOUD_COVER ≤ 30% was first applied, followed by pixel-level Quality Assessment (QA) masking of cloud, cloud shadow, and snow in the Level 2 products. Eighteen scenes remained after filtering; the mean cloud cover was 7.07% (ranging from 0 to 28.81%). To mitigate single-date bias and retain spectral contrast relevant to land degradation, both phenological periods were included: 15 scenes from the growing season (April to October) and 3 scenes from the dormant season (November to March). A temporal median composite was then generated from these 18 cloud-free images, which suppresses residual atmospheric effects and seasonal outliers while preserving stable spectral properties suitable for index calculations.

### Digital Elevation Model and pre-processing

The topographic skeleton of the study area was established using NASA SRTM1 (30 m) data (NASA Earthdata, 2013). The Digital Elevation Model (DEM) was re-projected to the WGS 84/UTM Zone 37 N coordinate reference system using bilinear interpolation with the gdalwarp utility in GDAL. Hydrological consistency was ensured using the FillDepressions algorithm in the WhiteboxTools v2.3 package (Lindsay, 2016) by following the flow direction D_8_ as the first step after filling the pits, followed by the Specific Catchment Area (SCA) and finally the Slope (*θ*). The resulting *θ* and *SCA* raster layers formed the input variables for the composite erosion and moisture indices (Hengl & MacMillan, 2019) .

### Length-Slope factor

The RUSLE-based Length-Slope (LS) factor was calculated using the Sediment Transport Index tool binary. The slope length *L* was calculated using Eq. (1) with the ratio of the slope length *L* to the reference length of *22.13* m and the slope steepness (sin *θ*) to *0.0896* (Gupta et al., 2023).

(1) \begin{eqnarray*}L\mathbf{S}=(L/22.13)^{m}X(\sin \nolimits ~\theta /0.0896)^{n}.\end{eqnarray*}

Here, the constants *m* = 0.4 and *n* = 1.3 are literature values for medium-slope field conditions.

*L* = (SCA −cell size) where cell size = 30 m (Desmet & Govers, 1996; Moore & Burch, 1986).

### Stream Power Index

The Stream Power Index (SPI) characterizes the combination of runoff intensity and slope and is calculated using Eq. (2) (Abdelkrim, Faid & Eslamian, 2024).

(2) \begin{eqnarray*}\mathbf{SPI}=A-\tan \nolimits ~\theta .\end{eqnarray*}

Here, A= is SCA×cellsize (m^2^) and *θ* = slope angle (rad). High SPI zones are susceptible to rill erosion and severe sediment transport (Šiljeg et al., 2023).

### Sediment Transport Index

To describe the topographic control of sediment load, the Stream Power Index (SPI) index was calculated using Eq. (3).

(3) \begin{eqnarray*}\mathbf{STI}=(A/22.13)^{m}-(\sin \nolimits ~\theta /0.0896)^{n}.\end{eqnarray*}

The STI equation uses *m* = 0.4 (Liu, Zhang & Xie, 2002) and *n* = 1.3 (Zhang et al., 2017). The equation has the same structure as RUSLE-LS, emphasizing potential sediment capacity instead of current strength.

The topography of Battalgazi Plain is dominated by medium slopes. For this slope class, the 221 standardized plot experiments on which RUSLE is based calibrated the length exponent *m* to 0.4, thus keeping the prediction error acceptable in agricultural areas with moderate slopes (Liu, Zhang & Xie, 2002). The coefficient *n* = 1.3 governing the steepness effect of slope is derived from log–log regression of more than 70 rainfall simulator studies and is included as a universal recommendation in the RUSLE2 science documentation (Zhang et al., 2017).

### Topographic Wetness Index (TWI)

The Beven-Kirkby wetness index, which indicates soil saturation tendency, was calculated using Eq. (4) (Beven & Kirkby, 1979).

(4) \begin{eqnarray*}\mathbf{TWI}=\ln \nolimits (A/\tan \nolimits ~\theta ).\end{eqnarray*}

In the equation, high values of A and low values of tan *θ* represent areas of spatial accumulation where water can accumulate and lead to saturation overflow.

The LS, SPI, STI and Topographic Wetness Index (TWI) rasters were observed to have minimum extreme values. These extreme negative values were considered to be indicative of system-induced NoData and were therefore excluded from the study by excluding all pixels with < 0 prior to analysis (Sørensen, Zinko & Seibert, 2006). Next, all topo-hydrographic rasters were min-max normalized as (*X*−*X*min)/(*X*max−*X*min), where *X* is the raw pixel value of the given index, and *X*min and *X*max are the minimum and maximum of that index within the study area after masking negative values. Normalization was performed separately for each raster (LS, SPI, STI, TWI), yielding dimensionless values in [0,1]. Subsequently, polygon means were computed for modeling (Nguyen et al., 2017). This methodology allowed topo-hydrographic parameters at different scales to be made comparable and integrated into machine learning models for land degradation assessments.

### Land degradation vulnerability calculation

A method based on the SIR was applied to quantitatively assess the vulnerability to land degradation. The SIR was adapted to assess the risk of land degradation. The LDV map was produced using the Geometric Mean Form of SIR (Eq. (5)). The identified key factors were weighted equally to produce a sensitivity score ranging from 0 to 1 for each location. Since the sensitivity map is based entirely on ground truth data (national land survey data), it was used as “ground truth”, *i.e.,* local reference data in the modeling studies. Remote sensing-derived spectral indices (*e.g.*, vegetation indices) and hydro-topographic indicators (*e.g.*, slope, topographic wetness index, *etc.*) were used as independent variables, and models were created to predict the values of the ground truth map. Taking the terrestrial map as a reference ensures that the model outputs reflect actual terrain conditions; in fact, integrating remote sensing-derived indicators with terrain data and topography is a common approach in land degradation risk analyses (D’Acunto, Marinello & Pezzuolo, 2024).

(5) \begin{eqnarray*}SI{R}_{LDV}={ \left( LCS\times WRBS\times SDCS\times ESS \right) }^{1/4}.\end{eqnarray*}

In Eq. (5), SIR_LDV_ denotes the geometric mean adaptation of the STORIE index combining four normalized, dimensionless component scores. LCS is land use capability class score, WRBS is major soil reference groups score, SDCS is slope and depth combination score, and EES is erosion severity score.

Combining the component scores with geometric mean also reflects the “limiting factor” principle that is characteristic of the SIR system; when the score of any factor is very low (*e.g.*, if the terrain is flat and there is minimal risk of erosion), the multiplicative structure also significantly reduces the overall index, strongly reflecting this favorable situation in the final result (Rossiter, 1995). Thus, even the weakest aspect of each site influences the overall sensitivity value, allowing a cautious and comprehensive approach to degradation risk assessment.

Land Use Capability has classes from I to VIII, with higher numbered classes referring to land with the lowest potential for agricultural use, usually due to severe constraints such as slope, soil depth, stoniness or erosion (Kouli, Soupios & Vallianatos, 2009). Higher land capability classes therefore indicate a higher risk of potential degradation (Du et al., 2015). Major Soil Groups are the main categories defined according to the morphological and physical characteristics of soils, and different soil types show varying vulnerability to erosion and degradation processes (FAO, 2006; Panagos et al., 2015). For example, shallowly developed or sandy-textured soils are considered more prone to degradation processes such as erosion and organic matter loss compared to deep-profile and well-structured soils (Aksoy et al., 2010). The combination of slope and soil depth, which is considered as the third component, involves the evaluation of the topographic conditions of the land together with the soil profile thickness. As slope increases, soil losss increases exponentially as the flow rate and carrying capacity of water increase (Nearing, 1997; Tao et al., 2022). This effect is particularly pronounced in areas where soil depth is shallow, *i.e.,* plant rooting depth and water holding capacity are limited (Luo et al., 2024). Therefore, slopes with steep gradients and shallow soils are considered to be the most susceptible areas to land degradation.

Erosion severity is the fourth component, which refers to the degree of erosion currently observed on the relevant land. Land with significant surface erosion or severe soil loss is indicative of current degradation and implies a high risk of future degradation. Indeed, sites that show signs of severe erosion receive high vulnerability scores, while areas with no or very mild erosion receive lower scores (Brahim et al., 2020; Tsymbarovich et al., 2020). Each component was converted into normalized scores in the range of 0–1 to represent vulnerability to land degradation. This scoring was implemented to reflect the relative risk level of the respective class or attribute, with the lowest risk conditions receiving a value of 0 (or very close to 0), and the highest risk classes receiving a value of 1 (or close to 1) (Table 2). For example, Class I land was assigned a score close to 0, while Class VII–VIII land was assigned a score close to 1. Similarly, fertile and deep-profile soil groups were assigned low scores (low risk), while groups with fragile or shallow soils were assigned high scores (high risk). All components were weighted equally in the analysis, meaning no single factor was prioritized over another.

**Table 2:** Criteria and scoring used in land degradation vulnerability calculation. Scoring criteria used to quantify land degradation vulnerability based on soil reference groups (WRB), USDA land capability classes, slope–depth combinations, and erosion severity. Scores were assigned according to relative susceptibility to degradation (Borrelli et al., 2017; Ferreira et al., 2022; Hartemink, 2006; Nearing, 1997).

**Criterion**	**Class / Category**	**Score**
**Soil Major Reference Groups (WRB)**		
	Fluvisols	0.5
	Cambisols	0.8
	Chromic Cambisols	0.7
	Colluvisols	0.6
	Luvisols	0.6
	Calcaric Luvisols	0.8
	Calcaric Cambisols	0.7
**USDA Land Capability Classes**		
	I	0.1
	II	0.2
	III	0.3
	IV	0.5
	VI	0.7
	VII	0.9
	VIII	1
**Slope-Depth Combinations**		
	Slope 0–2%, deep (>90 cm)	0
	Slope 0–2%, moderate (50–90 cm)	0.08
	Slope 2–6%, deep (>90 cm)	0.14
	Slope 2–6%, moderate (50–90 cm)	0.21
	Slope 6–12%, moderate (50–90 cm)	0.36
	Slope 6–12%, shallow (20–50 cm)	0.43
	Slope 6–12%, very shallow (0–20 cm)	0.5
	Slope 12–20%, deep (>90 cm)	0.42
	Slope 12–20%, moderate (50–90 cm)	0.5
	Slope 12–20%, shallow (20–50 cm)	0.57
	Slope 12–20%, very shallow (0–20 cm)	0.65
	Slope 20–30%, moderate (50–90 cm)	0.64
	Slope 20–30%, shallow (20–50 cm)	0.71
	Slope 20–30%, very shallow (0–20 cm)	0.79
	Slope >30%, shallow (20–50 cm)	0.85
	Slope >30%, very shallow (0–20 cm)	0.93
**Erosion Severity**		
	None or very slight water erosion	0.1
	Moderate water erosion	0.3
	Severe water erosion	0.7
	Very severe water erosion	0.9

All spatial analysis and calculations were performed using ArcGIS 10.8 software (Esri, Redlands, CA, USA). Data layers in different scales and formats were merged and overlaid in the GIS environment to obtain the relevant class values for each unit area. Then, predisposition scores in the range of 0–1 corresponding to each class were automatically assigned with the help of a mapping table, and geometric mean calculation was applied for each location using these scores. The resulting SIR values were subsequently mapped to visualize the spatial distribution of LDV.

### Land degradation vulnerability prediction method

This section describes the methodology for estimating the LDV index values determined in the previous section with the XGBoost regression model based on remote sensing and hydro-topographic indicators.

### Data and variables

The input variables of the model consist of spectral indices, which are derivatives of digital elevation models. Input variables, comprising these 11 components, were averaged for polygon vector data and an input dataset was prepared. LDV scores produced with the terrestrial dataset were used as the target variable (dependent variable). Before starting the analysis, various pre-processing steps were applied on the raw data. These steps aimed to prepare a suitable data set for modeling by removing possible inconsistencies in the data. Once all the data was prepared, the dataset was divided into training and testing to ensure model validation. As is common practice, the data is split by random sampling, with 70% of the data for training and 30% for testing (Acharya, Daigh & Oduor, 2021). The modeling unit was defined as a spatial polygon. The means of all covariates were aggregated per polygon using zonal statistics, and the LDV target variable was assigned to the same polygon (Khosravani et al., 2024). Stratified random splitting was applied to preserve the LDV distribution: 1.244 polygons (70%) were allocated for model training, and 534 polygons (30%) were retained as an independent external validation set. The validation set was not used during Optuna hyperparameter optimization. No polygon was included in more than one subset. The training dataset was used for model learning and hyperparameter optimization, while the remaining independent test dataset was used to evaluate the performance of the trained model on unseen data. During this separation, it was ensured that the target variable distribution was similar in both subsets to facilitate a fair model evaluation.

### Model development and hyperparameter optimization

**Figure 3:**
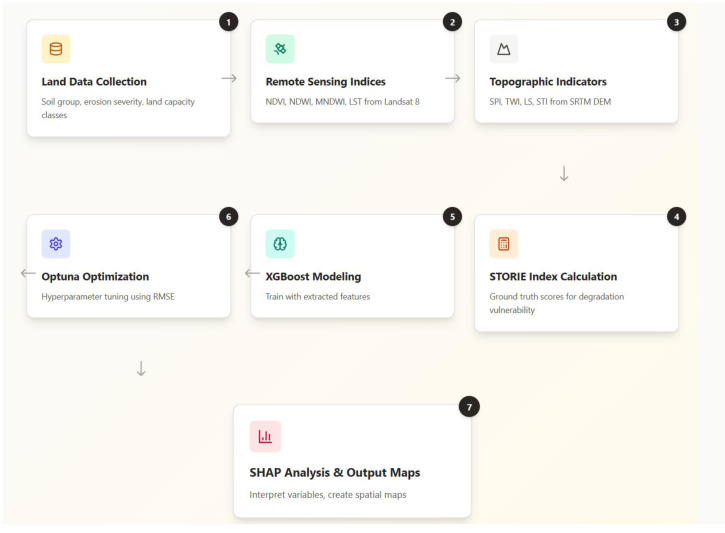
Methodological flowchart for modeling land degradation sensitivity with remote sensing and machine learning. Methodological flowchart illustrating the sequential framework used to model land degradation sensitivity. The process integrates ground-truth soil attributes, remote sensing indices (*e.g.*, NDVI, LS, SPI), and topographic variables with a machine learning pipeline based on Optuna-optimized XGBoost. Final outputs include LDV risk scores and explanatory SHAP values.

Following the data preprocessing steps, the XGBoost regressor model was used to predict LDV scores (Fig. 3). XGBoost is a gradient boosting machine learning algorithm based on an ensemble of decision trees. This algorithm is recognized for its ability to learn complex and nonlinear relationships with high computational efficiency and its capacity to prevent overfitting through regularization (Elith, Leathwick & Hastie, 2008; Yin et al., 2025). XGBoost, which gives successful results especially in large-sized and multivariate data sets, was also preferred in this study.

As the first stage of model development, the hyperparameter search space was identified. A comprehensive set of hyperparameters were optimized to maximize the performance of the XGBoost model. The main hyperparameters examined included learning rate, tree depth (max_depth), number of trees (n_estimators), subsample rate, feature subsampling rate (colsample_bytree) and regularization coefficients (reg_lambda, reg_alpha). Subsequently, a Bayesian optimization-based search was performed using the Optuna library to identify the optimal combination of parameters for the model in the defined hyperparameter search space. Optuna is a next-generation hyperparameter optimization framework with the flexibility to dynamically run experiments in a define-by-run approach (Akiba et al., 2019). In this process, the search algorithm, guided by the results from randomly initialized trials, probed combinations of hyperparameters in successive trials towards the optimal direction. In each Optuna trial, the model was built and validated with the relevant hyperparameters. To reliably measure the generalization ability of the model during hyperparameter optimization, 5-fold cross-validation (5-fold CV) was applied.

The training data was divided into five equal folds; for each trial, the model was trained five times with a different part as a validation set and with the remaining four parts. In this way, the average performance of each hyperparameter combination was calculated to mitigate the risk of overfitting. Optuna optimized based on the mean error (*e.g.*, Root Mean Square Error (RMSE) or performance score (*e.g.*, coefficient of determination (*R*^2^)) obtained after 5-fold CV as the target metric. Internal validation used 5-fold CV run exclusively on the 1.244 polygon training set during Optuna. Folds were built at the polygon level to prevent leakage. Each trial trained on four folds and validated on one fold; fold sizes were approximately 249, 249, 249, 249, and 248 polygons. The objective of optimization was to minimize the mean RMSE across the five validation folds. *R*^2^ was reported for reference and used only as a tie breaker when mean RMSE values were equal within tolerance. The final model was retrained on the full training set and evaluated once on the independent 534 of polygon test set. The best performing hyperparameter set was selected as the combination that provided the highest accuracy and lowest error in CV. Once the best hyperparameters were determined by Optuna, the XGBoost model was retrained with these optimal parameters on the entire training data. During training, care was taken to keep the model complexity under control by using XGBoost’s built-in regularization mechanisms and techniques such as early stopping (Pang et al., 2024). For early terminating, a criterion was applied such that training was stopped when no improvement was observed for 50 consecutive rounds on a small validation set separated from the training data. The final XGBoost regressor model, with its optimized hyperparameters, was ready to predict the LDV scores.

The error metrics RMSE, Mean Absolute Error (MAE), *R*^2^ and Huber Loss were used to quantitatively assess the performance of the XGBoost regressor model in predicting LDV scores (Table 3). RMSE and MAE measure the magnitude of error between model predictions and actual values; MAE treats all errors with equal weight, while RMSE penalizes large errors more (Chicco, Warrens & Jurman, 2021; Ramsubhag & Hosein, 2024). The *R*^2^ is a measure of how much of the variance in the dependent variable is explained by the independent variables and usually takes values between 0 and 1; the closer to 1, the greater the predictive power of the model (Chicco, Warrens & Jurman, 2021). Huber Loss is an error function that is more robust to outliers, which are common especially in environmental data; it balances the advantages of MAE and MSE by applying a quadratic penalty for small errors and a linear penalty for large errors (Huber, 1973). A low Huber Loss value indicates that the model is robust to outliers and the predictions are reliable (Ramsubhag & Hosein, 2024). These metrics have also been used to assess overfitting by comparing the performance of the model on training and test sets.

**Table 3:** Error metrics and formulas used in model performance evaluation. Note: *y*_*i*_, actual value; $\hat {{y}_{i}}$, predicted value; $\overline{y}$, mean of *y*; *n*, total number of samples; *δ*, delta threshold (for the Huber loss); *L*_*δ*_(*y*, *f*(*x*)), Huber loss function; and *f*(*x*), model output.

Name	Abbreviation	Formula	Reference
Root Mean Squared Error	RMSE	$\sqrt{ \frac{1}{n} {\mathop{\sum }\nolimits }_{i=1}^{n}{ \left( {y}_{i}-\hat {{y}_{i}} \right) }^{2}}$	Chai & Draxler (2014)
Mean Absolute Error	MAE	$ \frac{1}{n} {\mathop{\sum }\nolimits }_{i=1}^{n} \left\vert {y}_{i}-\hat {{y}_{i}} \right\vert $	
Coefficient of Determination	R^2^	$1- \frac{{\mathop{\sum }\nolimits }_{i=1}^{n}{ \left( {y}_{i}-\hat {{y}_{i}} \right) }^{2}}{{\mathop{\sum }\nolimits }_{i=1}^{n}{ \left( {y}_{i}-\overline{y} \right) }^{2}} $	Chicco, Warrens & Jurman (2021)
Huber Loss Function	Huber Loss	${L}_{\delta } \left( y,f \left( x \right) \right) = \frac{1}{2} { \left( y-f \left( x \right) \right) }^{2}\cdot {1}_{ \left\vert y-f \left( x \right) \right\vert \leq \delta }+ \left( \delta \left\vert y-f \left( x \right) \right\vert - \frac{1}{2} {\delta }^{2} \right) \cdot {1}_{ \left\vert y-f \left( x \right) \right\vert > \delta }$	Huber (1973)

Interpreting the predictions and variable effects of complex “black box” machine learning models such as XGBoost is indispensable for scientific credibility and for generating actionable results for management policies (Abdullah & Mustafa, 2025). Therefore, SHAP analysis was employed to elucidate the decision mechanisms of the model and to quantitatively assess the contribution (positive or negative impact) of each variable to the final prediction of the model (Abdulrashid et al., 2025). SHAP is a method inspired by game theory and measures the marginal contribution of each attribute to the prediction of the model (Pinichka et al., 2025). The SHAP analysis was utilized to understand the importance ranking, direction and magnitude of the variables in model estimation.

Focus was also placed on identifying potential areas of spatial uncertainty that reflect the spatial variation of the generalization ability of the model and may indicate systematic errors due to environmental heterogeneity (Chen, Shao & Zhang, 2024). Spatial uncertainty analysis was performed to assess the spatial reliability and precision of model predictions (Liu et al., 2025). As a complementary step to model performance evaluation and interpretation, it is crucial to understand the spatial reliability and variability of model predictions (Malik et al., 2025). By mapping the level of uncertainty in the spatial distribution of model outputs, this analysis reveals regions where forecasts are more reliable (low uncertainty) and regions that require careful interpretation (high uncertainty) (Zhao, Zhang & Shu, 2025). The spatial uncertainty map represents internal cross-validation variability, computed as the standard deviation of predictions across the five CV models (Eq. (6)) (Viana & Haftka, 2009). Spatial uncertainty was quantified as the standard deviation of the five predictions obtained for each soil mapping polygon during 5-fold cross-validation of the XGBoost model, with hyperparameters optimized *via* Optuna. Equivalently, spatial uncertainty values were derived by calculating the standard deviation of the predictions from the 5-fold CV ensemble used in the Optuna hyperparameter optimization process (Viana & Haftka, 2009; Chen & Guestrin, 2016; Akiba et al., 2019). For cartographic visualization, values were classified into five classes using the Natural Breaks (Jenks) classification method (Jenks & Caspall, 1971). This uncertainty metric reflects model ensemble variability arising from sensitivity to training data partitioning and does not encompass measurement errors or uncertainties from testing datasets (Lilburne et al., 2024; Rohmer, Belbeze & Guyonnet, 2024). Low uncertainty indicates that the model predictions are more consistent across different cross-validation folds in the region of interest, while high uncertainty indicates high variability in predictions.

(6) \begin{eqnarray*}{U}_{i}=sd({\hat {y}}_{i}^{(1)}{\hat {y}}_{i}^{(2)}{\hat {y}}_{i}^{(3)}{\hat {y}}_{i}^{(4)}{\hat {y}}_{i}^{(5)}).\end{eqnarray*}

In Eq. (6), *U*_*i*_ denotes the uncertainty value for polygon *i*; sd(⋅) denotes the standard deviation operator; ${\hat {y}}_{i}^{(k)}$ denotes the prediction for polygon *i* from the *k*th cross validation fold (*k*=1…5); *y*_*i*_ denotes the observed value for polygon *i*; when residual standard deviation is used, ${e}_{i}^{(k)}={y}_{i}$ −${\hat {y}}_{i}^{(k)}$ replaces ${\hat {y}}_{i}^{(k)}$ in the calculation.

## Results

### Radiometric and topo-hydrographic indices as input features

**Figure 4:**
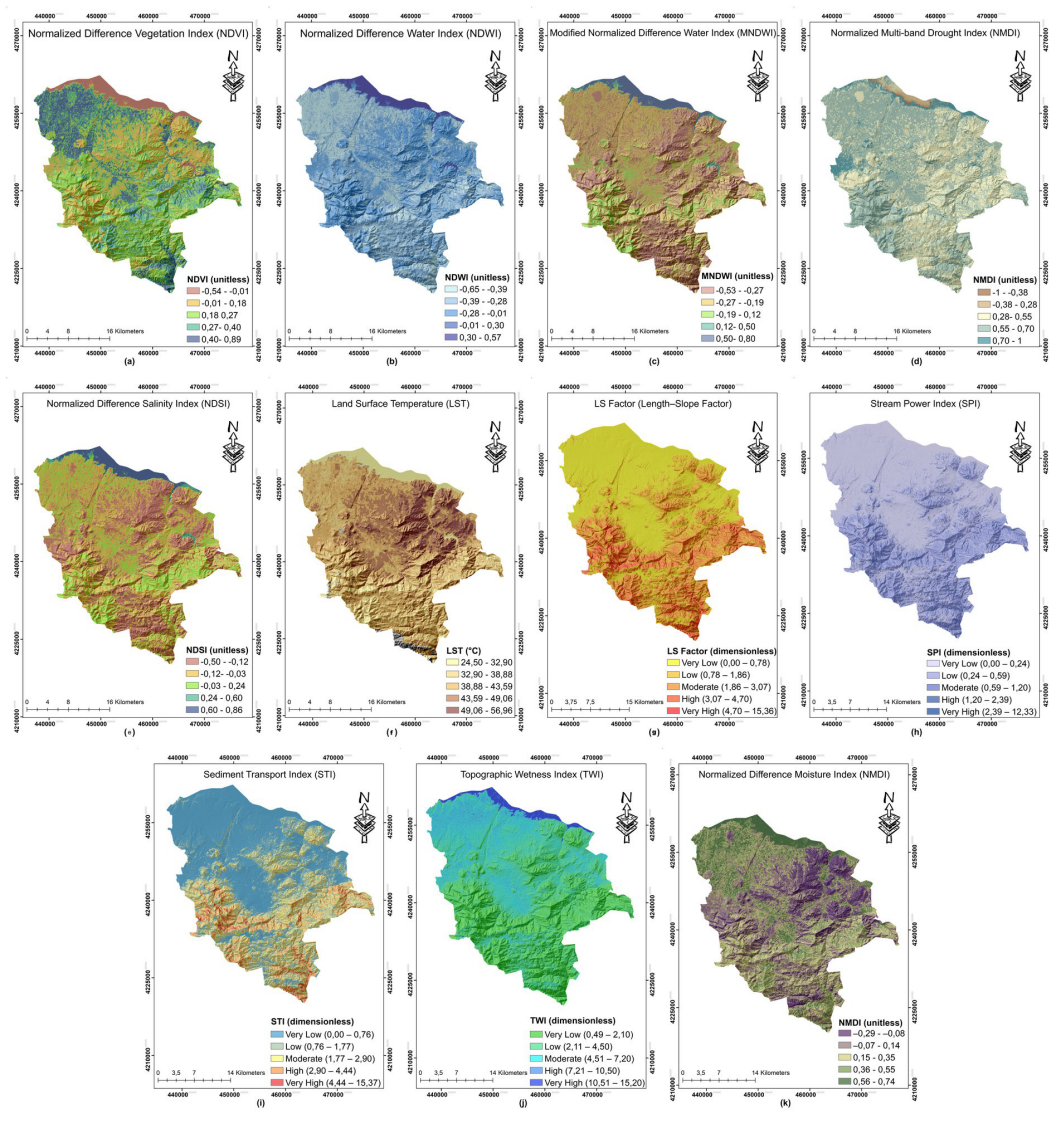
Remote sensing and topo-hydrology indices used as covariates. (A–K) The remote sensing and topo-hydrological indices used as covariates in the land degradation modeling framework. These include vegetation and moisture indices (*e.g.*, NDVI, Normalized Difference Moisture Index (NDMI), NMDI), surface reflectance-based indices (*e.g.*, NDWI, MNDWI), and terrain-derived variables such as Slope, LS Factor, SPI, STI, and TWI. All indices were resampled to 30 m resolution and harmonized spatially for pixel-level modeling. Raster sources: Landsat Collection 2 Level-2 surface-reflectance products from Landsats 5, 7 and 8 (USGS/NASA, public domain, https://www.usgs.gov/landsat-missions/landsat-collection-2). Digital elevation model: Shuttle Radar Topography Mission v3.0 DEM, 30 m (NASA/USGS, DOI 10.5066/F7PR7TFT, public domain, https://www.usgs.gov/centers/eros/science/usgs-eros-archive-digital-elevation-shuttle-radar-topography-mission-srtm).

Remote sensing indices used as covariates in LDV estimation are shown in Fig. 4. In the northern part, vegetation density is high with NDVI values higher than 0.5 (Fig. 4A) and Land Surface Temperature (LST) values lower than 25 °C (Fig. 4F). Surface-vegetation moisture is maintained with positive values of Normalized Difference Water Index (NDWI) and Modified Normalized Difference Water Index (MNDWI) (Figs. 4B and 4C) and Normalized Multi-band Drought Index (NMDI) moves into the positive range (Fig. 4D). In the central and southern belt, NDVI is lower than 0.2, while NDWI is below 0 and MNDWI is almost −0.10. LST exceeds the 35 °C threshold. NMDI below −0.10 confirms soil moisture deficiency. The salinity map (Fig. 4E) identifies core areas where Normalized Difference Salinity Index (NDSI) locally exceeds 0.30 in the northern marginal zone. In the middle plateau-south slope belt, LS and STI values higher than 10 (Figs. 4G and 4I) coincide with NDVI less than 0.20, NDWI less than 0, and LST higher than 35 °C. SPI (Fig. 4H) remains moderate, concentrating in localized valleys. In the northern elevations, NDVI > 0.50, NDWI > 0.30 and LST < 25 °C correspond with minimum levels of LS and STI. Threshold zones with TWI higher than 12 (Fig. 4J) concentrate in the downwind topographic hollows of the northern main ridge and on the main drainage floors, characterized by positive NDWI and MNDWI values. Arid upstream slopes with TWI lower than 4 on the south-eastern plateau edge couple with NMDI lower than −0.10 and LST above 35 °C. SPI values reach the highest levels in the mid-scale tributary-junction areas, and STI is also high in these sections.

### Model architecture and accuracy assessment

**Figure 5:**
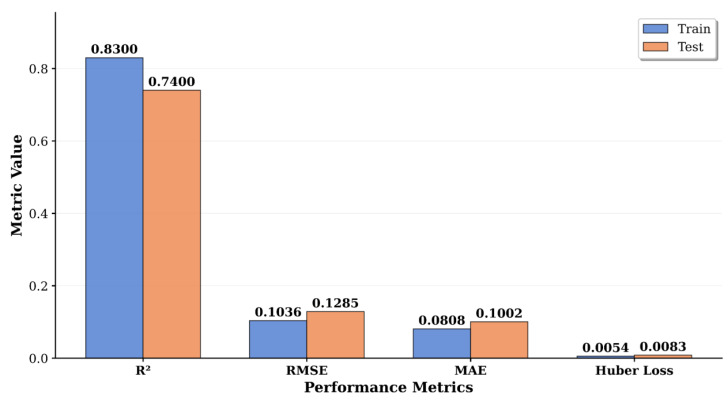
Training-versus-testing performance of the XGBoost regression model. Bar heights show the coefficient of determination (*R*^2^), root-mean-squared error (RMSE), mean-absolute error (MAE) and Huber loss obtained on the 70% training subset and the independent 30% test subset of the dataset (n = total samples; split performed with stratified random sampling). Numerical labels on each bar indicate the exact metric value. Higher *R*^2^ and lower RMSE, MAE and Huber loss indicate better predictive skill; thus the modest drop from train to test suggests limited over-fitting. *R*^2^, RMSE and MAE are dimensionless in this context because the target variable was min–max normalised prior to modelling. Huber loss was calculated with *δ* = 1. Abbreviations: *R*^2^, coefficient of determination; RMSE, root-mean-squared error; MAE, mean-absolute error. Colours: blue = training; orange = testing.

The XGBoost regression model developed for the prediction of LDV showed satisfactory performance in both the training and the test datasets. The RMSE value obtained in the training set was 0.1036, MAE value was 0.0808 and *R*^2^ value was 0.83. In the test set, RMSE value was 0.1285, MAE value was 0.1002 and *R*^2^ value was 0.74. In addition, the Huber Loss value of the model was 0.0083 in the test set (Fig. 5).

**Figure 6:**
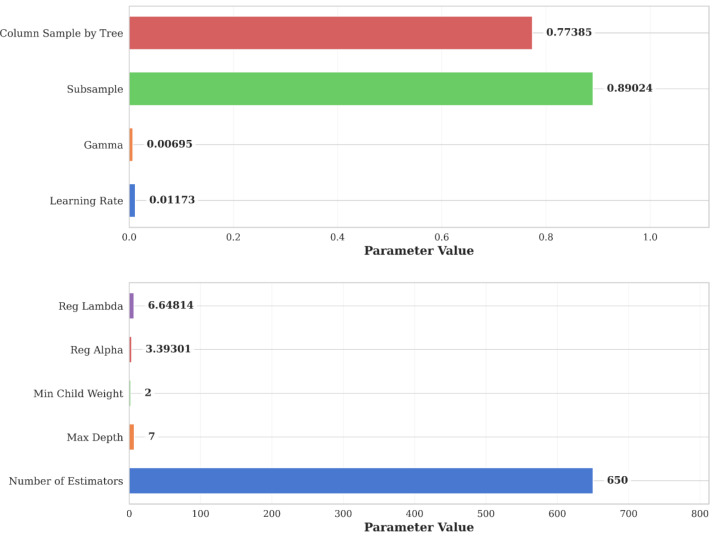
XGBoost hyperparameter values optimized with Optuna. Bar chart showing the optimal XGBoost hyperparameter values selected *via* Optuna optimization. Parameters include learning rate (eta), maximum tree depth, number of estimators, subsample ratio, column sampling, and regularization terms (lambda, alpha). These values yielded the best predictive performance during cross-validation.

The optimal hyperparameter values obtained using Optuna hyperparameter optimization are shown in Fig. 6. This combination of hyperparameters increases the model’s ability to capture complex environmental relationships while reducing the risk of overfitting. The difference in performance between the training and test sets (a decrease of 0.09 in *R*^2^ values) indicates that the model possesses a reasonable generalization ability and does not overfit. The increase in RMSE and MAE values (0.0249 and 0.0194, respectively) are similarly acceptable. This confirms that the model can make reliable predictions on the test data without overfitting the training data. The low Huber Loss value (0.0083) indicates that the model is robust to outliers and the predictions are reliable.

The XGBoost model explained about 74% of the variance of LDV in the test dataset (*R*^2^ = 0.74) and showed low error magnitudes (RMSE = 0.128, MAE = 0.100) (Fig. 5). The proximity of the MAE to the RMSE indicates that the errors are mostly medium-sized, while the exceptionally low Huber loss (0.008) confirms that outliers have minimal impact on overall performance. This result is also corroborated by the residual values.

**Figure 7:**
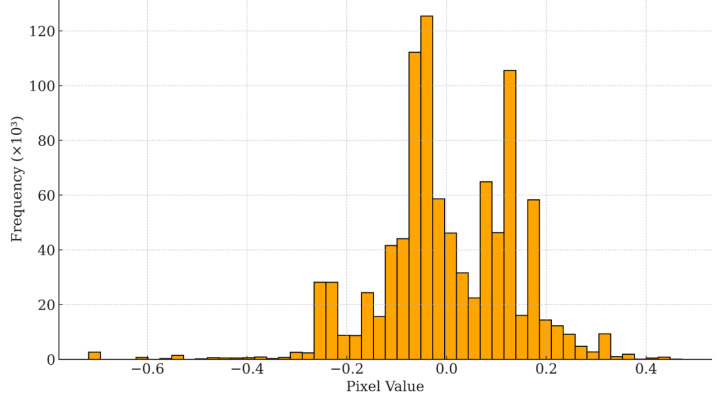
Histogram of spatial (raster-based) residuals of XGBoost model predictions. Histogram of spatial residuals between observed and predicted land degradation vulnerability values from the XGBoost model. Residuals were computed at the pixel level across the study area, with values centered around zero, indicating minimal spatial bias and good overall model calibration.

The Histogram analysis (Fig. 7) reveals that the residuals are concentrated in a narrow range (−0.06 ≤ *ɛ* ≤ 0.11), their mean value is almost neutral (0.003) and their distribution is limited (*σ* = 0.137). The quartile limits (Q_1_ = −0.061; Q_3_ = 0.089) confirm that more than 50% of the errors fall within this limited range. Despite the overall symmetry, the slight positive skewness (+0.427) indicated that small-scale overestimates were slightly more frequent. The extreme values (−0.718; +0.427) accounted for only 0.7 of the pixels and had a negligible impact on the RMSE.

**Figure 8:**
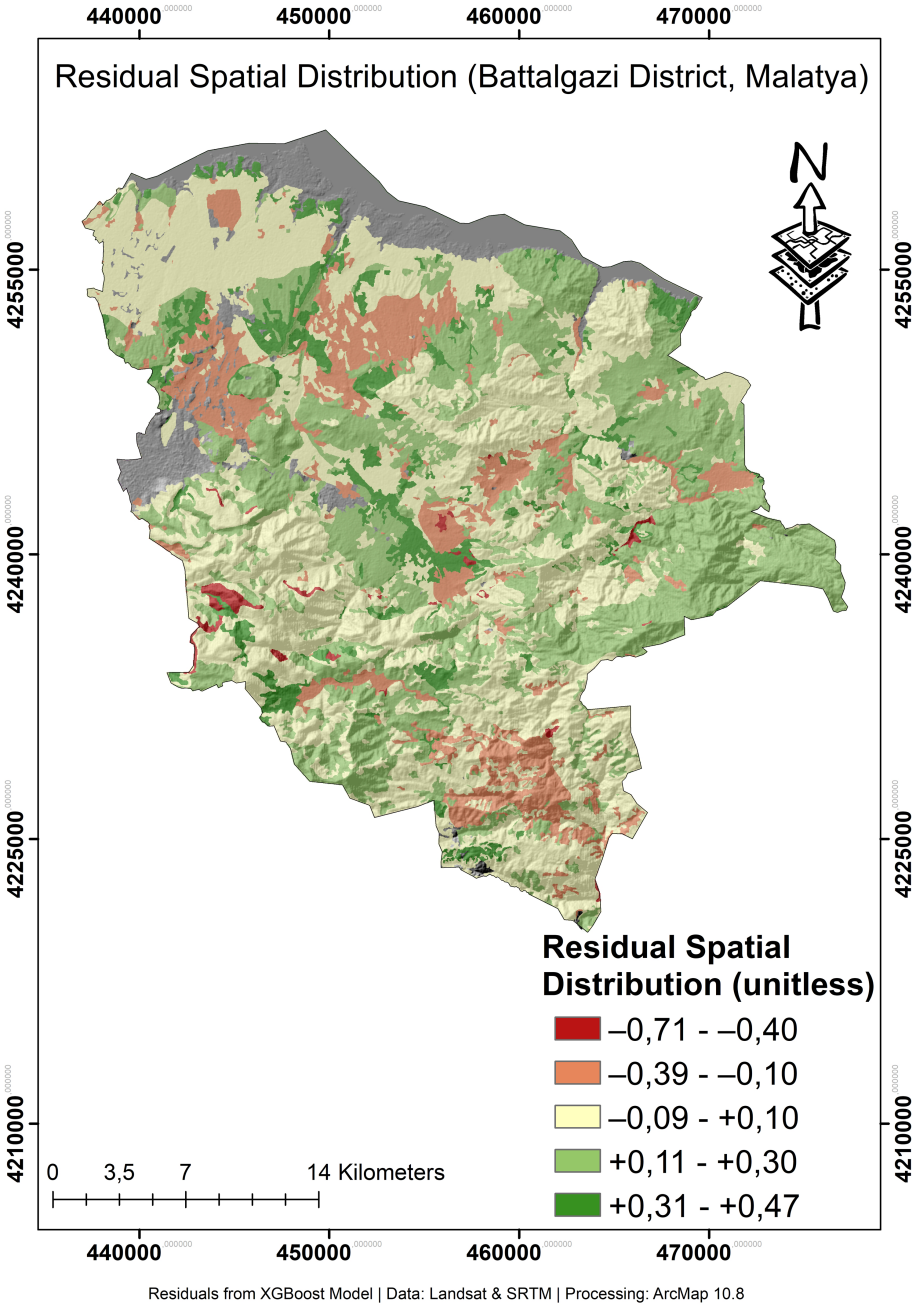
Spatial distribution of model residual values in the study area. Residual raster (*ɛ*): author-generated difference between the ground-truth STORIE-based LDV index and predictions from an Optuna-optimised XGBoost model at 30 m resolution. Positive values (yellow to red) denote over-prediction, negative values (purple to blue) denote under-prediction, and near-zero errors are shown in turquoise/cyan.

The residual map (Fig. 8) shows non-random spatial clustering, which contrasts with pure stochastic noise. While most areas exhibit minimal error (±0.05, shades of turquoise/cyan), certain systematic deviations appear. These areas are located in the southwest of the study area and appear as purple or blue on the map (*ɛ* < −0.25). In these areas that consistently produce low estimates, there are likely unaccounted factors influencing land degradation sensitivity that are not included among the model input variables. Conversely, the areas in yellow and red (*ɛ* > 0.25) in the northeastern part of the study area produced systematic overestimation. Here the model’s limitations were evident and the influence of missing inputs, possibly related to groundwater or intensive irrigation effects, is likely here. The low RMSE and MAE and the narrow histogram kernel are consistent with the large regions of low error (turquoise) in the map (Fig. 8). Conversely, the clustered deviations of the residual map and the positively sloping tail of the histogram reveal local limitations. Importantly, the clusters of spatial under/overestimates are directly related to specific environmental factors do not present in the model, as shows. The error metrics and residual distribution map show that high accuracy is achieved, evidenced by the corresponding residual distribution, but systematic regional errors persist due to unresolved environmental heterogeneity.

### SHAP analysis

**Figure 9:**
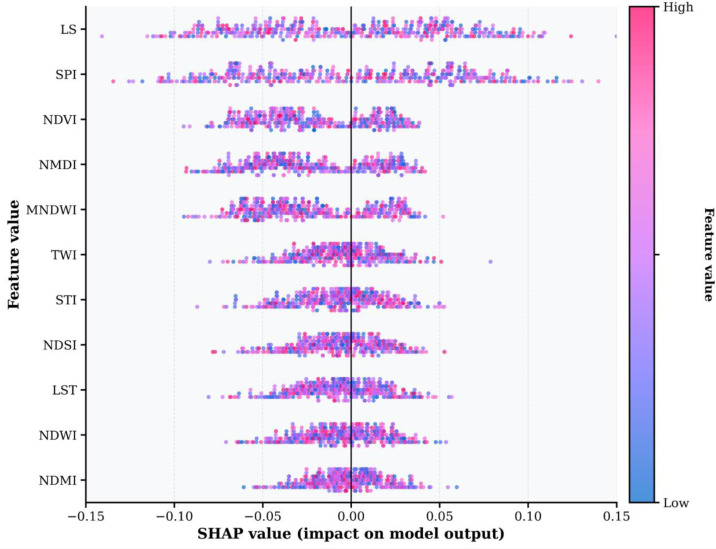
SHAP summary plot of feature contributions to the land-degradation-vulnerability model. SHAP summary plot showing feature contributions to land degradation vulnerability predictions. Each dot represents a pixel; SHAP values on the *x*-axis indicate the direction and strength of influence, while colors reflect scaled feature values (pink = high, blue = low). Features are ranked by importance, with LS, SPI, and NDVI as top contributors.

SHAP analysis was used to elucidate the decision mechanisms of the Optuna-optimized XGBoost model and to quantitatively assess the contribution of each variable to model predictions. The importance of ranking and impact levels of the variables affecting the LDV index are shown in the beeswarm plot in Fig. 9. Variables are ranked from top to bottom according to their average absolute contribution to the model output. Each point represents a sample and the *x*-axis shows the SHAP value. Negative values indicate effects that reduce LDV, while positive values indicate effects that increase it. The color of the dots represents the original value of the variable, with blue indicating low values and pink indicating high values.

According to the SHAP feature importance ranking, topographic variables such as LS and SPI are the most decisive in prediction performance (Fig. 9), followed by spectral indices such as NDVI and NMDI. Hydro-topographic variables (LS, SPI, TWI, STI) produce higher SHAP values than spectral indices. LS and SPI have a wide range between −0.10 and +0.12, while NDVI, NMDI and MNDWI range within ±0.07. The SHAP effects of NDSI, LST, NDWI and NDMI show more limited impacts, generally remaining below ±0.05.

The LS is the most impactful variable, with SHAP values yield ranging from −0.10 to +0.12; high LS values (pink dots) typically yielded positive effects, while low values (blue dots) results in negative effects. SPI ranks second, showing a similar SHAP range and pattern. NDVI, the third most influential spectral index, has SHAP values ranging from approximately −0.07 to +0.07, in the case of NDVI, low NDVI values (blue dots) generally produce positive effects (Fig. 9).

### Spatial distribution of land degradation vulnerability

**Figure 10:**
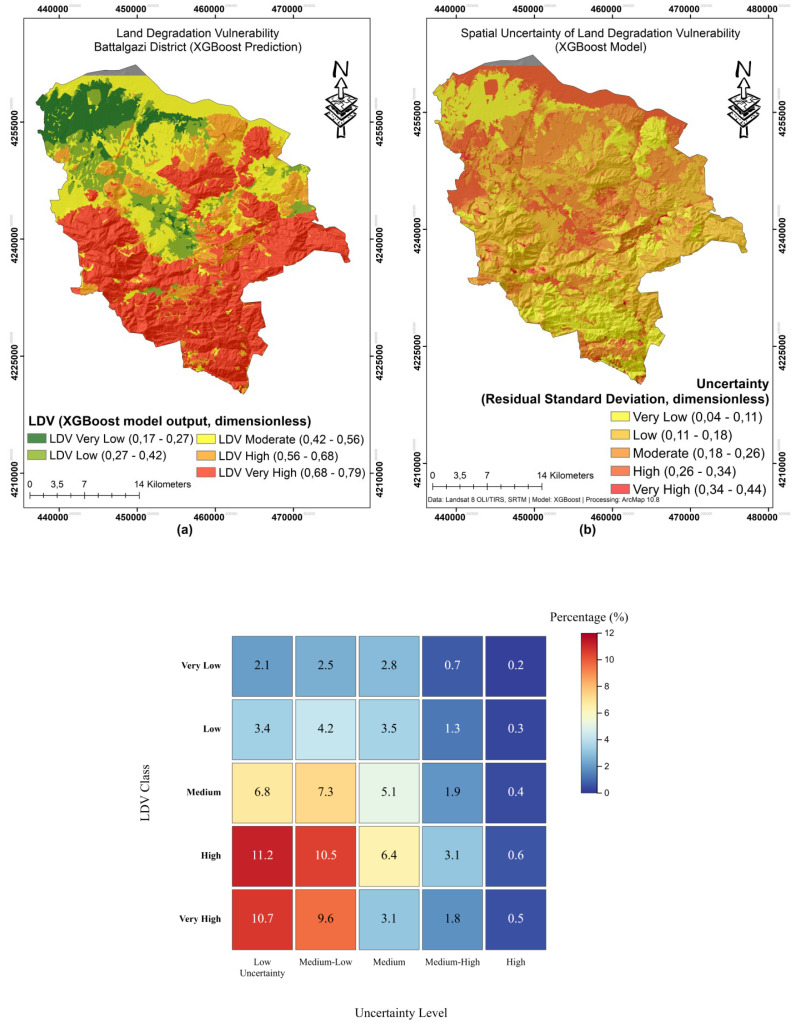
Predicted land-degradation vulnerability, spatial uncertainty, and class distribution in Battalgazi, Türkiye. (A) The predicted land degradation vulnerability (LDV) map generated using the XGBoost model. (B) Spatial uncertainty in the LDV predictions, highlighting areas with higher prediction variance. (C) A heat map of LDV class distributions across different uncertainty levels, supporting risk-informed interpretation.

The LDV map shows the percentage distribution of soil vulnerability to degradation in the study area (Fig. 10). Based on XGBoost model outputs and Natural Breaks (Jenks) classification, five LDV classes were defined: very low (0.17–0.27), low (0.27–0.42), medium (0.42–0.56), high (0.56–0.68) and very high (0.68–0.79), reflecting normalized vulnerability values between 0 and 1 m (Fig. 10A). Very low and low vulnerability are concentrated in the north and northwest, where vegetation is denser and soils are deeper. Moderate LDV appeared in the central transition zones. Conversely, high and very high LDV concentrated in the south and southeast, marked by steep slopes, shallow soils, sparse vegetation, and high runoff. The southeast exhibited the highest vulnerability (0.68–0.79).

The spatial uncertainty map shows confidence levels in LDV estimates, (Fig. 10B) Using standard deviations from 5-fold cross-validation, uncertainty was classified into five categories: low (0.04–0.12), medium-low (0.12–0.20), medium (0.20–0.28), medium-high (0.28–0.36) and high (0.36–0.44). About 68.3% of the area falls within low and medium-low uncertainty (0.04–0.20). Cross-tabulation (Fig. 10C) shows that in the high LDV class, 11.2% of pixels have low and 10.5% have medium-low uncertainty. In the very high LDV class, 10.7% have low and 9.6% have medium-low uncertainty. The very low LDV class is associated mainly with medium (2.8%), medium-low (2.5%) and low (2.1%) uncertainty. The low LDV class has 4.2% medium-low, 3.5% medium, and 3.4% low uncertainty. Moderate LDV areas show the widest range of uncertainty.

## Discussion

### Radiometric & topo-hydrographic features

The contrasting conditions between northern and southern regions demonstrate clear spatial polarization in ecosystem resilience. Northern areas with high NDVI and positive moisture indices create an ecological buffer zone that limits land degradation risk, supporting findings by Verhoeve et al. (2021) on vegetation resilience in similar environments. However, the long-term sustainability of this buffering effect under increasing temperatures remains uncertain.

The inverse relationship between vegetation cover and thermal stress in central-southern regions indicates accelerated degradation processes. Specifically, low NMDI values (<−0.10) combined with high LST confirm moisture deficiency patterns. This observation is consistent with Khan, Khan & Khan (2023) and Sharma et al. (2022), who demonstrated strong correlations between NMDI and soil moisture. Furthermore, the NDSI exceeding 0.30 in northern marginal zones suggests secondary salinization risk. This finding aligns with Kalambukattu et al. (2023)’s observations of increased NDSI in low-elevation areas.

The coincidence of high LS and STI values with sparse vegetation and elevated temperatures in the middle plateau-south slope belt reveals the synergistic effects of topographic and climatic factors on erosion processes. This correlation confirms the results reported by Borrelli et al. (2017) on erosion risk intensification on long, steep slopes with minimal vegetation cover. The concentration of runoff power in localized valleys, as indicated by moderate SPI values, demonstrates how topographic factors channel erosion processes and supporting (Moore, Grayson & Ladson, 1991).

The northern elevations function as an “upper watershed buffer” where favorable biophysical conditions combine with minimal topographic stress, validating Lal (2001)’s concepts of watershed-level buffer zones. High TWI zones in topographic hollows act as low-sensitivity micro-basins where moisture storage supports biological productivity, consistent with Li et al. (2020). Conversely, areas with TWI < 4 coupled with extreme thermal and moisture stress exhibit accelerated degradation cycles, supporting the findings of Malav et al. (2022) on vulnerability patterns in arid conditions.

The spatial distribution of SPI and STI values reveals critical erosion-deposition dynamics within the channel network. High values at tributary junctions indicate enhanced sediment transport capacity (Bizzi & Lerner, 2015). Where these zones intersect with degraded terrestrial conditions, a feedback cycle emerges between lateral sediment supply and channel instability (Sharma, 2010). In contrast, stream reaches with dense vegetation maintain channel stability through energy absorption and limited sediment mobilization. This observation confirms findings by Laonamsai et al. (2023) and Chen et al. (2024) on the role of vegetation in reducing sediment transport.

### Model architecture and accuracy assessment

The XGBoost model demonstrates robust predictive capability with *R*^2^ values of 0.83 (training) and 0.74 (testing), indicating reasonable generalization without severe overfitting. The moderate performance gap between training and test sets confirms the model’s ability to make reliable predictions on unseen data. Furthermore, the low Huber Loss value (0.0083) particularly indicates robustness to outliers, critical for environmental data applications where outliers are common.

Current model performance aligns closely with recent machine learning applications in soil property prediction. Li et al. (2025), for instance, reported an *R*^2^ of 0.82 for soil lead prediction using a hyperspectral imaging with XGB-Boruta-PCC algorithm. This result is comparable to the training performance (*R*^2^ = 0.82) reported in this study. While their dedicated feature selection improved accuracy, similar results were obtained in the current study without explicit feature selection, suggesting potential for additional gains by incorporating feature selection procedures.

The hyperparameter configuration reveals important optimization strategies for complex environmental data. The n_estimators value (650) is higher than the typical ranges reported by Yu et al. (2025), which may indicate more complex relationships among the input variables considered in this study. This was balanced by a conservative learning_rate of 0.0117, substantially lower than Li et al. (2025)’s optimal value of 0.064 for soil salinity prediction. The combination of high estimators with low learning rate promotes better generalization and model stability, corroborated by the acceptable performance gap between training and testing (Chen & Guestrin, 2016).

The max_depth value of 7 provides an optimal balance between model complexity and generalization ability. This value is consistent with the depth of 6 determined by Hasanuzzaman & Shit (2025) for similar applications. This parameter selection effectively mitigates the risk of underfitting caused by shallow trees and the overfitting caused by excessively deep trees, thereby contributing to the model’s robust performance on both the training and test datasets.

### SHAP analysis

The interpretability of machine learning algorithms through SHAP analysis is critical for environmental sustainability and land management applications. The transparency and explainability of model outputs are indispensable for scientific credibility and generating applicable results for policy makers and land managers (Gavade & Gavade, 2025; Sinha et al., 2025).

The dominance of topographic variables, particularly LS and SPI, in determining LDV aligns with fundamental erosion processes. The LS factor’s wide SHAP value range (−0.10 to +0.12) demonstrates its critical role as a main component of the RUSLE model, determining erosion potential as established by Wischmeier & Smith (1978). The high LS values increase surface flow velocity and kinetic energy, accelerating soil loss on steep and long slopes. This result is consistent with the findings of Arabameri et al. (2018), who also reported LS as the most influential factor in land degradation.

Recent studies provide valuable context for interpreting the SHAP results. For example, a decline in the slope-length-steepness (LS) factor has been shown to reduce erosion rates in agricultural lands, indicating that LS is not only a risk indicator but also an actionable tool for reducing erosion (Singh et al., 2025). In Ethiopia’s Abaya-Chamo Basin, LS values exceeding the recommended range of 2–18 t ha^−^^1^ year^−^^1^ were associated with annual soil loss of 1,445.73 t ha^−^^1^ year^−^^1^, highlighting the significant impact of this factor (Alemu et al., 2025). Additionally, the effects of LS should be evaluated not only as biophysical parameters but also in conjunction with Sustainable Development Goals (Samuel et al., 2025).

The SPI emerges as a dominant explanatory variable because it measures the erosion power of surface flow. High SPI values during heavy rainfall translate into greater sediment transport capacity and naturally coincide with valley floors and channel junctions susceptible to degradation (Moore, Grayson & Ladson, 1991). SPI also plays an important role in landslide susceptibility analyses, ranking among the most influential topographic factors (Conforti et al., 2014).

The advantages of SHAP analysis are not limited to identifying the order of importance of covariates; the analysis also enhances decision-making processes related to land management by visualizing the direction and intensity of their effects (Chen et al., 2025a). The detailed information provided highlights the necessity of implementing protective practices such as terracing, contour farming, and strip cropping, especially in steep areas with high LS values. Moreover, terracing and vegetation cover can reduce soil erosion by 60–70% on slopes with a gradient of over 15% (Panagos et al., 2015). Therefore, the SHAP-based results provide a concrete roadmap for designing and prioritizing targeted land protection strategies.

### Spatial distribution of land degradation vulnerability

The spatial distribution of LDV reveals distinct geographical patterns linked to environmental conditions. The presence of very low and low vulnerability in the northern and northwestern regions coincides with denser vegetation cover and deeper soils, confirming the protective role of these factors against degradation processes. This spatial pattern is consistent with previous SHAP analysis results which showed negative SHAP values for high NDVI conditions.

The concentration of high and very highly vulnerable areas in the south and southeast reflects the combination of multiple degradation factors such as steep slopes, shallow soils, sparse vegetation cover, and high surface runoff potential. These areas, which reach maximum vulnerabilities (0.68–0.79), represent critical regions requiring urgent conservation interventions. The spatial pattern confirms the SHAP results that high LS and SPI values have strong positive effects on vulnerability to degradation.

The spatial uncertainty map shows the level of confidence in LDV estimates (Fig. 10B). Uncertainty values range from 0.04 (low uncertainty) to 0.44 (high uncertainty). Most of the study area (68.3%) have low and medium-low uncertainty values (0.04–0.20). This indicates that LDV estimates have high reliability in most of the study area. Spatial uncertainty values were obtained by calculating the standard deviation of the predictions produced by the XGBoost model during the 5-fold cross-validation process. This approach is a widely used method to assess the spatial distribution of uncertainty in model predictions. Uncertainty values are categorized into five classes: low (0.04–0.12), medium-low (0.12–0.20), medium (0.20–0.28), medium-high (0.28–0.36) and high (0.36–0.44). This classification was performed using the Jenks natural refraction method (Lu, Cavieres & Moraga, 2023; Elia et al., 2023). Areas with high uncertainty values are generally concentrated in the northwestern parts of the study area, especially in areas with complex topographic features. These areas are generally characterized by heterogeneous land use, complex geomorphological structures, and variable soil properties. Areas with low uncertainty values are generally concentrated in the central and southern parts of the study area, especially in areas with soil characteristics. These areas generally represent regions with more consistent model predictions and likely a denser distribution of reliable observation points.

## Conclusions

This study presented the HyStoRSM (Land survey + Remote Sensing + Machine Learning) framework for mapping LDV in semi-arid regions. Aiming to address critical limitations of existing approaches, such as the inability of spectral indices to directly reflect subsurface constraints and microtopographic hydrological controllers, under-representation of the influence of topographic factors on erosion vulnerability, and gaps in the integration of field data and remote sensing data, this framework combines field-derived edaphic factors (soil depth, large soil group, erosion severity) with topographic-hydrological indices and radiometric remote sensing data using Optuna-optimized XGBoost models. The findings support the hypothesis that this hybrid approach can improve prediction accuracy and produce transparent factor attribution maps suitable for management scenarios. In particular, Optuna-based systematic hyper-parameter optimization and quantification of the effects of factors at the pixel level *via* SHAP outputs successfully addressed the lack of reproducibility and transparency in previous studies. This innovative approach provides an important reference point for proactive land degradation risk management in water-limited environments. Such comprehensive and interpretable maps will provide decision-makers with critical information to develop more effective and targeted strategies to combat land degradation, directly contributing to food security and protection of ecosystem services.

The findings of this study provide a solid foundation for future research. Testing the applicability of the HyStoRSM framework in different semi-arid regions will increase the generalizability of the model. Furthermore, a better understanding of land degradation dynamics through the integration of time series analyses and modeling of future risks under climate change scenarios will further advance knowledge in this field. This integrated and transparent modeling approach is a valuable contribution to global efforts to combat land degradation.

##  Supplemental Information

10.7717/peerj.20606/supp-1 Supplemental Information 1 Spectral, hydro-topographic and field-surveyed soil attributes for land-degradation vulnerability modelling in Battalgazi District, Türkiye This dataset contains 6534 spatial samples on a 30 m grid within the Battalgazi Basin. Each record includes eight Landsat-8 spectral indices (NDVI, NDMI, NDWI, MNDWI, NMDI, NDSI, STI, TWI), two SRTM-derived hydro-topographic factors (LS and SPI), and three field-measured soil variables. The target column provides the Land-Degradation Vulnerability (LDV) score derived via the HyStoRSM workflow.
• Field-derived variables: Land-Use Capability class, Major Soil Group, Slope–Depth combination, Erosion severity, Current land use, plus their STORIE scores.
• Topo-hydrological indices (derived from SRTM DEM): Stream Power Index, Length-Slope Factor (LS), Sediment Transport Index (STI), Topographic Wetness Index (TWI).
• Spectral/Landsat-8 indices: NDVI, NDMI, MNDWI, NDWI, NMDI.
• Surface conditions: Land-surface temperature (LST).
• Target variable: continuous land-degradation vulnerability (LDV) STORIE score.
These data were used for Optuna-tuned XGBoost training, validation, SHAP interpretation, and Figures 5–10. Missing values are flagged as “NA,” and no rows were excluded. Sharing this file enables complete reproducibility of our analyses.

10.7717/peerj.20606/supp-2 Supplemental Information 2 Python workflow for STORIE soil rating prediction via Optuna-tuned XGBoost and geospatial automation This script automates the entire STORIE score-modelling pipeline in Google Colab. It:
ingests a zonal-statistics shapefile containing 11 spectral and hydro-topographic predictors;
imputes, scales and caps outliers, then splits data 70 / 30;
tunes XGBoost hyper-parameters with Optuna (100 trials) and evaluates performance with *R*^2^, RMSE, MAE, MBD, Huber Loss and more;
generates SHAP explainability plots, feature-importance figures and 30 m resolution GeoTIFFs for predictions, residuals and uncertainty;
All code was written and tested solely by the corresponding author; variable names and directory paths can be adjusted in the header comments.

## Additional Information and Declarations

### Data Availability

The following information was supplied regarding data availability: The code and raw data is available in the Supplemental Files.
